# Melatonin Promotes Yield Increase in Wheat by Regulating Its Antioxidant System and Growth Under Drought Stress

**DOI:** 10.3390/biology14010094

**Published:** 2025-01-18

**Authors:** Xue Li, Jia Liu, Cuiping Zhang, Ze Liu, Xiang Guo, Shaoxiang Li, Hongsheng Li, Kun Liu, Kunzhi Li, Mingliang Ding

**Affiliations:** 1 Faculty of Life Science and Technology, Kunming University of Science and Technology, Kunming 650500, China; lixue@xtbg.ac.cn (X.L.); lz74648360@163.com (Z.L.); 2 CAS Key Laboratory of Tropical Plant Resources and Sustainable Use, Xishuangbanna Tropical Botanical Garden, Chinese Academy of Sciences, Menglun 666303, China; 3 Tuber Crops Research Institute, Yuxi Academy of Agricultural Sciences, Kunming 650204, China; jia@126.com (J.L.); yxnkyzcp@163.com (C.Z.); 4 Faculty of Environmental Science and Engineering, Kunming University of Science and Technology, Kunming 650500, China; guoxianglaw@163.com; 5 Food Crops Research Institute, Yunnan Academy of Agricultural Sciences, Kunming 650204, China; lsxnky@163.com (S.L.); lhsyaas@163.com (H.L.); ynliukun@126.com (K.L.); 6 Department of Plant Pathology, College of Plant Protection, China Agricultural University, Beijing 100107, China

**Keywords:** wheat, melatonin seed coating, antioxidant activity, yield

## Abstract

Drought stress constitutes a major challenge to wheat production. Melatonin plays a vital role in plants’ resistance to drought stress. Nevertheless, the influence of melatonin seed coating on the drought resistance ability of wheat remains unclear. Hence, in this study, wheat (Yunmai 112) was chosen as the experimental material. The research results indicated that 100 µM exogenous melatonin treatment enhanced the germination rate of wheat seeds by 11% compared to the CK group. Melatonin seed coating (100 or 200 µM) significantly inhibited the accumulation of ROS in wheat seedlings under drought stress conditions and facilitated the growth of wheat seedlings. Then, 100 µM melatonin seed coating elevated the activity of antioxidant enzymes (CAT, Cu/Zn-SOD, POD, and T-GSH) in wheat seedlings and strengthened the resistance of wheat to drought stress. In contrast to the control, 100 or 200 µM melatonin seed coating significantly raised the contents of soluble protein and chlorophyll in wheat seedlings. Further studies demonstrated that 100 µM melatonin seed coating promoted the increase in the thousand-grain weight and yield of wheat under drought stress. Taking together, melatonin seed coating is an effective approach for enhancing the stress resistance and the yield of wheat under drought stress.

## Simple Summary

In this study, wheat was selected as the experimental material. Our results discovered that under drought conditions, melatonin seed coating could reduce oxidative damage by enhancing the activities of antioxidant enzymes, as well as the contents of soluble sugar, soluble protein, and chlorophyll in wheat seedlings. Further investigations revealed that melatonin seed coating facilitated the increase in the thousand-grain weight and yield of wheat under drought stress. Briefly, melatonin coating is an effective approach for enhancing the stress resistance and yield of wheat under drought stress.

## 1. Introduction

Wheat (*Triticum aestivum* L.) is an annual or biennial gramineous herbaceous plant. It is the world’s third-largest crop after maize and rice [1]. Throughout the protracted evolution of agriculture, wheat has emerged as one of the most extensively distributed, produced, high-yielding, and nutritionally rich food crops globally, with around one-third of the global population depending on it as their primary sustenance [2]. Meanwhile, wheat has a wide range of uses, including as animal feed, human food, and industrial raw materials [3,4]. As the population keeps growing, the demand for wheat is increasing annually, and it is estimated that the demand for wheat will rise by approximately 28% in 2025 [5]. As one of the main food crops and sources for humans in the world, the stable growth of wheat yield is crucial [6].

However, adverse environmental factors, drought stress, heavy metals, and salt stress seriously affect the growth, development, and yield of wheat [7,8,9]. Drought stress severely limits the growth and yield of food crops. With the global climate gradually warming, this problem is becoming more and more serious [10]. The areas designated for wheat cultivation frequently experience varying levels of drought, and the resultant water scarcity significantly impairs both the growth and productivity of wheat. Consequently, improving the drought resilience of wheat is crucial for wheat food security [11]. Drought during the flowering stage of wheat will lead to aberrant growth and even total crop failure. Drought during the maturity phase of wheat will substantially diminish its yield [12]. Furthermore, drought induced by high temperatures shortens the growth cycle of wheat, causing smaller wheat seeds and substantial yield reduction [13].

In recent years, numerous studies revealed that plant hormones exert significant regulatory effects on the growth, maturation, and yield of wheat [14]. Melatonin, also known as N-acetyl-5-methoxytryptamine, is a small molecule substance that is highly evolutionarily conserved and is gradually being recognized as a potential novel plant hormone [15]. Dubbel and Hattori first discovered the trace of melatonin in higher plants in 1995, laying the foundation for subsequent research on plant melatonin [16,17]. With the continuous deepening of research, it was found that melatonin acts as a signaling molecule and participates in a variety of plant processes, such as growth, development, flowering, and fruiting [18]. Another study demonstrated that melatonin participates in the physiological processes of plants, including primary and secondary metabolism, the manufacture of plant hormones, and the improvement in crop output and quality [19]. Additionally, melatonin can also increase the resistance of plants to disadvantageous environmental conditions, for example, high temperature, drought, cold, and salt stress [20].

Reactive oxygen species (ROS) serve as essential signaling molecules in plants. When plants experience stress, reactive oxygen species (ROS) can rapidly accumulate, leading to membrane lipid peroxidation, DNA damage, protein degradation, and cellular autophagy [21]. Melatonin is regarded as a free radical scavenger and a powerful antioxidant, which is very important for protecting cells from oxidative damage caused by reactive oxygen species (ROS) and reactive nitrogen species (RNS) [22]. It has been reported that melatonin not only promotes plant growth and enhances the efficiency of photosynthesis and the content of chlorophyll but also reduces the formation of ROS, thereby protecting plants from oxidative stress [23]. And it also plays an important role in fruit development and delaying aging [24]. Melatonin was confirmed to enhance the antioxidant capacity by reducing the ROS content in apple, tomato, and wheat [25,26,27]. In drought stress, melatonin preserves the bioactivity of sugars and proteins in apples and grapes by mitigating chlorophyll breakdown and damage to the photosystem [28]. Melatonin can elevate proline, amino, and organic acids to safeguard wheat seedlings against cold stress [29].

These functions of melatonin render it extremely significant in agricultural production. However, it remains unclear whether seed coating with different concentrations of melatonin can enhance wheat’s resistance to drought stress. Therefore, in this study, different concentrations of melatonin were added to the coating of wheat seeds to explore the effects of melatonin on the germination rate, growth, antioxidant capacity, and yield of wheat.

## 2. Materials and Methods

### 2.1. Production of Wheat Seed Coating

The seeds of wheat (Yunmai 112) were provided by Food Crops Research Institute, Yunnan Academy of Agricultural Sciences, Kunming, China, Yuxi Academy of Agricultural Sciences, Yuxi City, Yunnan Province, China. This wheat variety is characterized by its good drought tolerance and high yield potential, and it is currently the most widely cultivated dryland wheat variety in Yunnan Province, China. The seeds of wheat were the currently predominant wheat variety in Yunnan Province of China. As the formula for the wheat seed coating involves a patent that is about to be published, we are temporarily unable to provide the detailed seed coating formula. According to the ratio of 1 mL of seed coating agent per 100 g of seeds, add melatonin to the corresponding volume of seed coating agent to make the final concentration of melatonin 0, 25, 50, 100, and 200 μM, respectively. After the wheat seed coatings with different concentrations of melatonin are prepared, they are dried and stored for subsequent wheat sowing.

### 2.2. Plant Growth and Drought Treatment

The wheat seed coating prepared in 2.1 was sown in the experimental field of the Jiajing experimental farm (1615.2 m above sea level, 102°17′52″ E, 24°10′3″ N) of the Yuxi Academy of Agricultural Sciences in Yanhe Town, Yuxi City, Yunnan Province, on 25 October 2023. The planting area of each treatment plot was 6.67 m^2^ (4.09 m × 1.63 m), the row spacing was 0.233 m, and there were 7 rows. Each treatment had three replicates and was randomly arranged. The physical and chemical properties of the soil in the planting area are shown in Table S1. The air temperature variation throughout the growth period of wheat was shown in Table S2. Throughout the growth period, no artificial irrigation and no water was applied throughout the whole growing period of wheat in the experimental field. As the wheat planted in this experiment was winter wheat, during the wheat growth season, the average precipitation in Hongta District, Yuxi City, Yunnan Province, was only 37.7 mm, with frequent strong winds causing significant soil moisture evaporation, and a severe drought occurred. The total rainfall during the entire growth period was far below the water requirement for normal wheat growth, so the wheat was under drought stress throughout the growth period. The wheat was harvested on 19 April 2024 after it matured. The wheat seeds were dried in the sun and then used for subsequent experimental testing.

### 2.3. Seeds Germination

Healthy seeds of approximately the same size were manually selected and sterilized in 1% sodium hypochlorite (*w*/*v*) for 15 min. Then, they were washed 7 times with sterile distilled water. Post-sterilization, the surface moisture of these seeds was absorbed with sterile filter paper, and the seeds were dried. Next, these seeds were allocated to petri dishes with varying concentrations of melatonin (0, 25, 50, 100, and 200 µM) and incubated in a light incubator at approximately 25 °C, with 65% humidity, a light intensity of 100 μmol m^−2^ s^−1^, and a photoperiod of 16 h light and 8 h dark for 60 h. The germination rate was counted every 12 h. Each treatment contained 30 seeds, and there were 3 replicates for each treatment.

### 2.4. Measurement of Fresh Weight, Plant Height, and Root Length

One month after sowing, the wheat treated with different concentrations melatonin seed coating entered the tillering stage. Healthy wheat seedlings about one month old were collected and photographed. The dirt surrounding roots of wheat seedlings was meticulously rinsed under running water, minimizing harm to roots as much as feasibly possible. The fresh weight, plant height, and root length of the wheat seedlings treated with different concentrations of melatonin seed coating were measured, respectively. For each treatment, 10 seedlings were used, and each treatment was replicated three times.

### 2.5. Measurement of the Contents of ROS, MDA, and Proline

Fresh one-month-old wheat seedlings were selected, 0.1 g of wheat seedlings were ground into powder, and then the samples were lysed with the extraction solutions corresponding to the content determination kits of H_2_O_2_, superoxide anion (O_2_^•−^), MDA, and proline, respectively. The obtained supernatants were used for the determination of the contents of the above four substances. The H_2_O_2_ content was determined using a H_2_O_2_ assay kit (Nanjing Jiancheng, Nanjing, China, A064-1-1) based on spectrophotometry. The content of superoxide anion (O_2_^•−^) was determined using the O_2_^•−^ assay kit (Nanjing Jiancheng, A052-1-1) based on colorimetry. The content of MDA was determined using the MDA assay kit (Nanjing Jiancheng, A003-1-2) based on TBA. The content of proline was determined using the proline assay kit (Nanjing Jiancheng, A107-1-1) based on the ninhydrin method. For each treatment, 0.1 g plant material was used, and each treatment was replicated three times.

### 2.6. Determination of Antioxidant Enzyme Activities

The antioxidant enzyme activity was determined by grinding 0.1 g of fresh one-month-old wheat seedlings with 1 mL of PBS solution (pH 8.0). The homogenate was centrifuged at 12,000× *g* for 10 min at 4 °C, and the supernatant was collected to determine the activities of CAT, Cu/Zn-SOD, POD, and T-GSH. The activities of CAT were measured using the assay kit (Nanjing Jiancheng, A007-1-1) based on the ammonium molybdate method. The activities of Cu/Zn-SOD were measured using the assay kit (Nanjing Jiancheng, A001-2-1) based on the hydroxylamine method. The activities of POD were measured using the Assay Kit (Nanjing Jiancheng, A084-3-1) based on colorimetry. The activities of T-GSH were measured using the assay kit (Nanjing Jiancheng, A061-1-1) based on the micro-enzyme labeling method. For each treatment, 0.1 g plant material was used, and each treatment was replicated three times.

### 2.7. Determination of Soluble Protein, Sugar, and Total Chlorophyll Content in Wheat Seedlings

Fresh one-month-old wheat seedlings were selected, and a 0.1 g sample was swiftly frozen in liquid nitrogen and fully ground extract with 1 mL of the extraction solution. The soluble sugar content was determined using the instructions provided in the assay kit (Nanjing Jiancheng, A145-1-1) based on the anthrone colorimetric method. The method for determining soluble protein content was similar to that for soluble sugar, and the content of soluble protein in the sample was measured using the soluble protein assay kit (Nanjing Jiancheng, A045-1-1) based on the biuret method. The determination of chlorophyll content was carried out as described [30]. The total chlorophyll content was calculated as chlorophyll *a* and chlorophyll *b*. For each treatment, 0.1 g of plant material was used, and there were three replicates for each treatment.

### 2.8. Measurement of Individual Grain Weight, Thousand-Grain Weight, and Yield After Wheat Maturity

After wheat matured, the wheat was harvested on 19 April 2024, and random samples of wheat from different concentrations of melatonin seed coating were selected, with 30 samples in each group. The weight of each individual grain was measured separately, and the average value was calculated in which the total individual grain weight was divided by 30. For the different concentrations of melatonin seed coating group, 1000 grains of wheat seed were randomly selected and weighed on an electronic balance, and the thousand seed weight was expressed in grams. The planting area of each group was 6.67 m^2^, and the weight of wheat harvested from each group was divided by 6.67 to obtain the unit of kilograms per m^2^, which was then converted to kilograms per hectare as the unit of wheat yield. Each treatment had three replicates.

### 2.9. Determination of Related Quality Indicators After Wheat Maturity

Wheat from different concentrations of melatonin seed coat groups was harvested on 19 April 2024, dried in the sun. The protein content (%), wet gluten content (%), hardness value (%), flour yield (%), stability time (min), and setting time (min) of wheat were determined by using a DA7200 near-infrared analyzer (Boteong Company, Shenzhen, China). Each treatment had three replicates.

### 2.10. Statistical Analysis

Data represent means ± SD of three replicates. Different in letters indicate significant differences according to Fisher’s least significant difference (LSD) test (*p* < 0.05)

## 3. Results and Discussion

### 3.1. Melatonin Promotes Germination Rate

**Figure 1:**
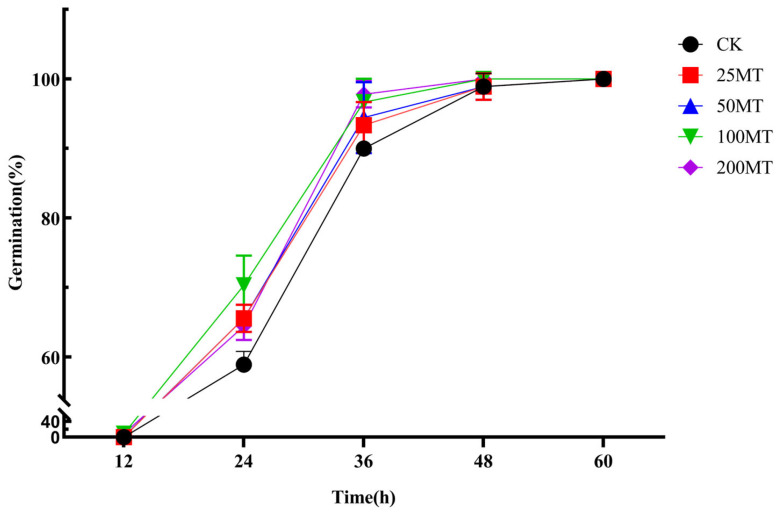
Effect of different melatonin concentrations on germination rate of wheat seeds.

As shown in Figure 1, there was essentially no germination in wheat seeds within the initial 12 h. It was discovered that different concentrations of melatonin all facilitated seed germination compared to CK at 24 h. Compared with the CK group, germination rates were increased by 7%, 7%, 11%, and 5%, among which the highest germination rate occurred when the melatonin concentration was 100 µM. When the incubation time was prolonged to 36 h, germination rates in melatonin groups remained higher than those in CK, with increases of 3%, 4%, 7%, and 7.8% compared with CK group, respectively. At 48 h, there was no difference in germination rates between melatonin groups and CK. Upon extending incubation period to 60 h, all seeds exhibited germination, achieving a germination rate of 100%. In summary, melatonin promotes the germination of wheat seeds, and the promoting effect t was best when the melatonin concentration is 100 µM.

### 3.2. Melatonin Promotes Wheat Seedling Growth Under Drought Conditions

**Figure 2:**
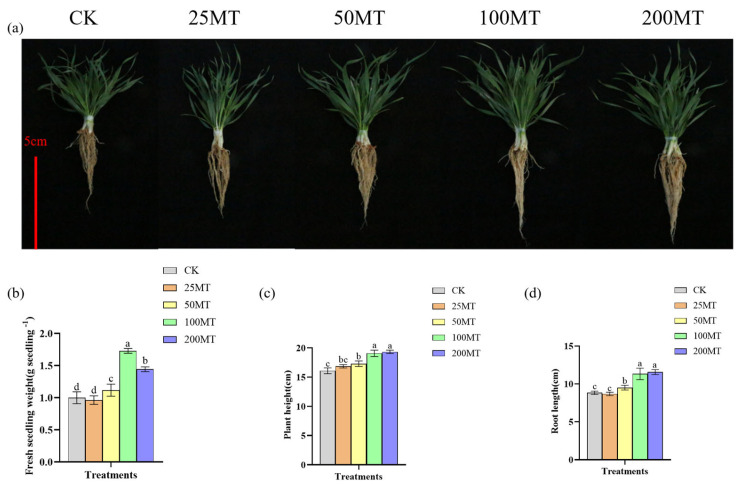
Effect of different melatonin concentrations on the growth of wheat seedlings under drought conditions. (**a**) one-month-old wheat; (**b**) effect of melatonin on the fresh weight of wheat plants; (**c**) effect of melatonin on the height of wheat plants; (**d**) effect of melatonin on the root length of wheat plants. Bar = 5 cm. Data were expressed as mean ± SD. Significant difference was represented different lowercase letters (*p* < 0.05).

Seed coating is an innovative method for improving agricultural yield and harvests. This is due to its ability to manage pests and diseases, stimulate growth and development, and improve agricultural yields. One-month-old wheat seedlings were collected in the field and photographed. Under drought conditions compared with CK group, adding different concentrations of melatonin in the wheat seed coating promoted the growth of wheat (Figure 2a). As the concentration of melatonin increased in wheat seed coating, the fresh weight of wheat exhibited an increased trend. The promotion effect was optimal when melatonin seed coating was 100 µM, with a fresh weight of approximately 1.8 g per plant (Figure 2b). Similarly, compared to the CK group, the plant height of wheat seedlings was the highest, reaching 19.2 cm when melatonin seed coating was 100 or 200 µM (Figure 2c). Likewise, when melatonin seed coating was 100 or 200 µM, the root length of the wheat seedlings was the longest, reaching 11.5 cm (Figure 2d). Therefore, the above experimental results indicated that 100µM melatonin seed coating plays a significant role in promoting the growth of wheat under drought conditions.

### 3.3. Melatonin Reduces the Oxidative Damage in Wheat Seedlings Under Drought Conditions

**Figure 3:**
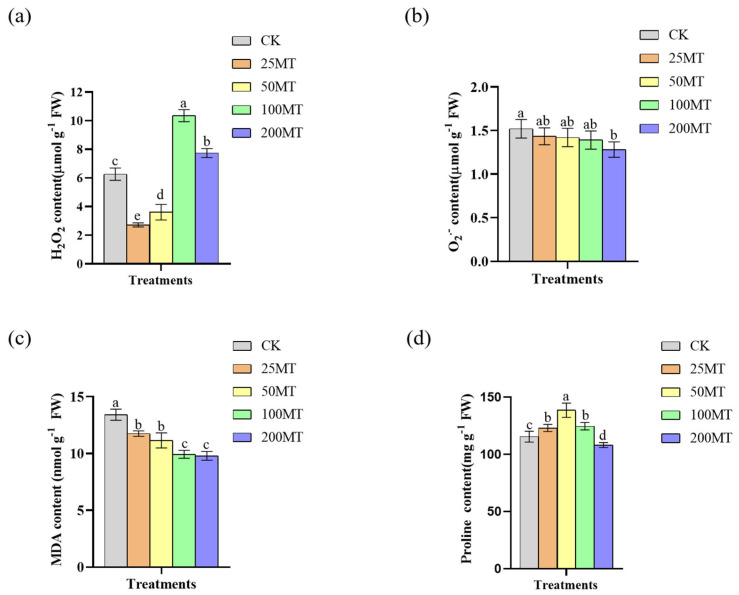
Effect of different melatonin on the content of H_2_O_2_, O_2_^•−^, MDA, and proline of wheat seedlings under drought conditions: (**a**) the content of H_2_O_2_; (**b**) the content of O_2_^•−^; (**c**) the content of MDA; (**d**) the content of proline. Data were expressed as mean ± SD. Significant difference was represented different lowercase letters (*p* < 0.05).

Oxidative damage was estimated by measuring the contents of H_2_O_2_, O_2_•^−^, MDA, and proline. Under disadvantaged conditions, plants accumulated a significant amount of reactive oxygen species (ROS), which can exert adverse effects on the growth and development of plants. It is clear that melatonin is considered a scavenger of H_2_O_2_, but in this study, we found that high concentrations of melatonin also induce H_2_O_2_ production. Under drought conditions compared to CK, low concentrations of melatonin seed coating suppressed the generation of H_2_O_2_ in plant. However, higher concentrations of melatonin (above 100 µM) induced the production of H_2_O_2_ in plant (Figure 3a), and this might be related to the fact that melatonin can both eliminate H_2_O_2_ and induce its production. When melatonin seed coating was 200 µM, the generation of O_2_^•−^ was significantly inhibited only 1.2 µmol g^−1^ compared to CK (Figure 3b). Similarly, 100 or 200 µM melatonin seed coating significantly inhibited the production of MDA compared to CK, and the content of MDA was least, at 9.8 nmol g^−1^ (Figure 3c). The content of proline reflects the plant’s resistance to adverse conditions to some extent. It can be known from Figure 3d that when melatonin seed coating was 50 µM, the content of proline was highest, reaching 138.6 mg g^−1^. When the concentration of melatonin seed coating gradually increases, the content of proline slowly decreases. This might be because the high concentration of melatonin activates other signaling pathways in wheat, thereby inhibiting the accumulation of proline. In brief, an appropriate melatonin seed coating significantly reduces the oxidative damage of wheat seedlings under drought conditions.

### 3.4. Melatonin Increases Antioxidant Activity in Wheat Seedlings Under Drought Conditions

**Figure 4:**
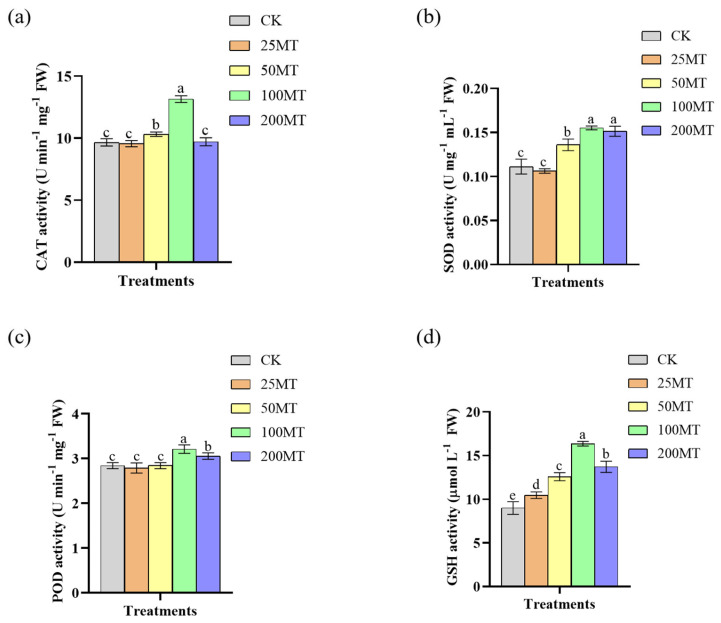
Effect of different concentrations melatonin on the activity of antioxidant enzyme of wheat seedlings under drought conditions. (**a**) the activity of CAT; (**b**) the activity of Cu/Zn-SOD; (**c**) the activity of POD; (**d**) the activity of GSH. Data were expressed as mean ± SD. Significant difference was represented different lowercase letters (*p* < 0.05).

In the antioxidant system of plants, CAT, Cu/Zn-SOD, POD, and T-GSH play a vital role. They work together to convert reactive oxygen species into harmless substances, thus protecting cells from oxidative damage. Under drought conditions compared with CK, when melatonin seed coating was 100 µM, the CAT enzyme activity was at its peak, reaching 13.15 U min^−1^ mg^−1^ (Figure 4a). Subsequently, CAT activity decreased with the increase in melatonin seed coating, indicating that a high concentration of melatonin would induce the production of H_2_O_2_ and thereby inhibit CAT activity (Figure 4a). When melatonin seed coating was 100 µM, the activity of SOD enzyme reached its maximum value, which was 0.16 U mg^−1^ mL^−1^ (Figure 4b). Similarly, low level melatonin seed coating failed to raise the POD activity. Only when melatonin was 100 µM, the POD activity was the highest, 3.21 U min^−1^ mg^−1^ (Figure 4c). Melatonin seed coating also significantly induced the T-GSH activity, and when melatonin seed coating was 100 µM, the GSH activity was at its highest, 16.36 µmol L^−1^ (Figure 4d). Thus, it can be inferred that 100µM melatonin seed coating significantly induces the antioxidant capacity of wheat seedlings under drought conditions.

### 3.5. Melatonin Improves the Content of Total Soluble Protein, Soluble Sugar, and Chlorophyll Under Drought Conditions

**Figure 5:**
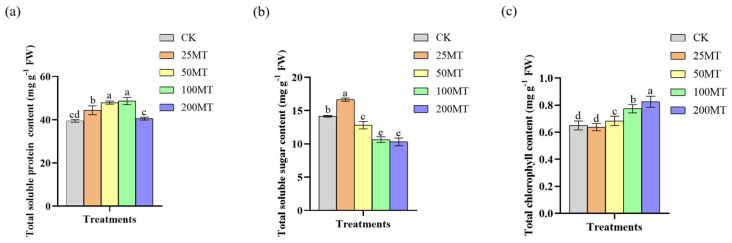
Effect of different concentrations melatonin on the content of soluble protein, soluble sugar, and chlorophyll of wheat seedlings under drought conditions. (**a**) the content of soluble protein; (**b**) the content of soluble sugar; (**c**) the content of chlorophyll. Data were expressed as mean ± SD. Significant difference was represented different lowercase letters (*p* < 0.05).

Our results showed that the soluble protein content was positively correlated with melatonin seed coating concentration, with the highest soluble protein content being 48.69 mg g^−1^, observed at 100 µM melatonin seed coating, increased by 9.2% compared to CK group (Figure 5a). However, only 25 µM melatonin seed coating promoted the increase in soluble sugar content, which increased by 2.5% compared to the CK group (Figure 5b). It indicated that high-level melatonin seed coating is not good for the accumulation of soluble sugar of wheat seedlings under drought stress conditions. Chlorophyll, an essential photosynthetic pigment in plants, plays a crucial role in the photosynthesis of plants. The total chlorophyll content significantly increased as melatonin seed coating increased, and it reached the maximum 0.83 mg g^−1^ at 200 µM melatonin seed coating (Figure 5c). Thus, it can be concluded that high-level melatonin seed coating promotes the increase in soluble protein and chlorophyll contents in wheat seedlings; on the contrary, low-level melatonin seed coating is good to the content of soluble sugar in wheat seedlings under drought stress conditions.

### 3.6. Melatonin Enhances the Thousand-Grain Weight and Yield of Wheat Under Drought Conditions

**Figure 6:**
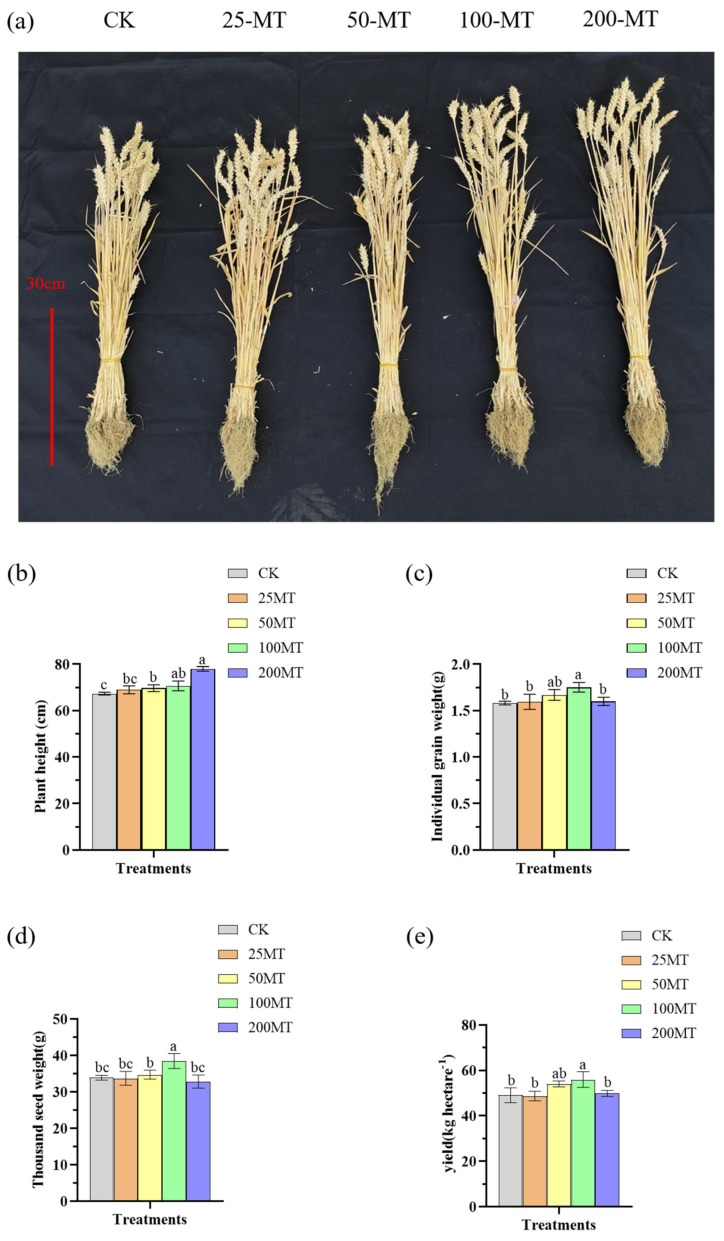
Effect of different concentrations melatonin on thousand-grain weight and yield of wheat under drought conditions. (**a**) the photograph of wheat after its maturity; (**b**) the plant height of wheat; (**c**) the individual grain weight of wheat; (**d**) the thousand seed weight of wheat; (**e**) the yield of wheat. Bar = 30 cm. Data were expressed as mean ± SD. Significant difference was represented different lowercase letters (*p* < 0.05).

Melatonin seed coating significantly promoted the height of wheat after maturity under drought conditions, and the increasing trend became more obvious as the concentration of melatonin seed coating gradually increased (Figure 6a). When melatonin seed coating was 200 µM, the height of wheat was the highest, 78 cm, and it was increased by 11% compared with CK group (Figure 6b). Additionally, 100 µM melatonin seed coating significantly promoted the increase in the individual grain weight, which increased by 2% compared to the CK group (Figure 6c). The thousand-grain weight is one of the crucial indicators for assessing the yield of wheat. This study discovered that low-level melatonin seed coating failed to promote the increase in the thousand-grain weight compared to the CK group (Figure 6d). When melatonin seed coating was 100 µM, which significantly promoted the increase in the thousand-grain weight under drought conditions, increasing by approximately 5% compared to the CK group (Figure 6d). Similarly, compared to the CK group, it can be observed that 100 µM melatonin seed coating also significantly promoted the increase in wheat yield by approximately 7% (Figure 6e). Taken together, when the melatonin seed coating was 100 µM, it significantly promoted the increase in individual grain weight, thousand-grain weight, and yield of wheat after maturation under drought conditions.

### 3.7. Melatonin Improves the Quality of Wheat After Its Maturity Under Drought Conditions

**Figure 7:**
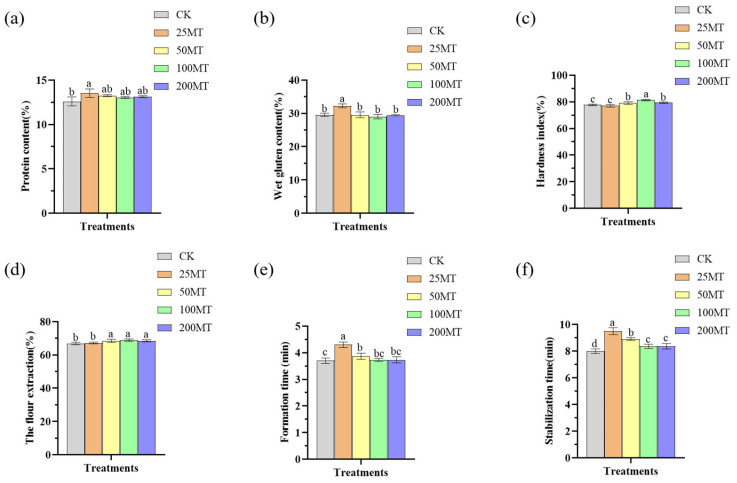
The effects of different concentrations melatonin seed coating on the quality of wheat after maturity under drought conditions. (**a**) the content of protein; (**b**) the content of wet gluten; (**c**) the hardness index; (**d**) the flour extraction; (**e**) the formation time; (**f**) the stabilization time. Data were expressed as mean ± SD. Significant difference was represented different lowercase letters (*p* < 0.05).

The wheat in different melatonin seed coating groups was harvested after maturity, and six quality indicators, including protein content, wet gluten content, hardness index, flour yield, formation time, and stability time, were determined using a Porton DA7200 near-infrared analyzer (Boteong Company, Shenzhen, China). Melatonin seed coating enhanced total protein accumulation in wheat grains under drought conditions, with optimal effects observed at 25 µM compared to CK (Figure 7a). Likewise, when melatonin was 25 µM, it significantly enhanced the wet gluten content in wheat grains relative to CK group (Figure 7b). Similarly, 25 µM melatonin seed coating was the most beneficial to the formation time (Figure 7e) and stability time (Figure 7f). From the above results, it can be inferred that under drought conditions, low-level melatonin seed coating had the best effect on the content of total protein, wet gluten, formation time, and stability time of wheat. However, high-level melatonin seed coating (100µM) markedly enhanced the hardness index of wheat grains (Figure 7c) and flour yield (Figure 7d) compared to CK. This result was the same as that of the thousand-grain weight and yield, indicating that the hardness value and flour yield rate are positively correlated with the wheat yield. Based on the above results, it can be concluded that, under drought conditions, low-level melatonin seed coating had best effects on the content of total protein, wet gluten, formation time, and stability time of wheat grains. High-level melatonin seed coating enhanced the hardness index of wheat grains and flour yield, ultimately increasing wheat yield.

## 4. Discussion

Drought is defined as a long-term absence of rain or low rainfall combined with high temperatures in a specific region, causing an extreme deficiency of water in the air and soil [31]. Drought is among the most severe disasters affecting humanity worldwide, characterized by high occurrence frequency, long duration, and extensive coverage [32]. Drought significantly impacts human society and results in substantial losses in agricultural productivity [33]. Nevertheless, numerous regions where wheat is cultivated are susceptible to extreme drought, thereby leading to a significant decline in wheat production [34]. Therefore, it is crucial to investigate methods for improving the drought resilience of wheat.

As a potential plant hormone, melatonin plays an important regulatory role in the growth and yield of plants [35,36,37]. In this study, we discovered that 100 µM exogenous melatonin treatment improved the germination rate of wheat seeds compared to the CK group at 24 and 36 h in a short duration (Figure 1). As early as 2019, Khan et al. discovered that the exogenous melatonin treatment significantly enhanced the germination rate of rapeseed seeds compared with the control group [38]. Based on the above experimental results, we inferred that melatonin, as a potential phytohormone, might facilitate seed germination by regulating the gibberellin signaling pathway. The results of field experiments also indicated that 100 µM melatonin seed coating significantly promoted the increase in plant fresh weight, plant height, and root length (Figure 2). More and more studies showed that melatonin, as a pleiotropic signaling molecule, plays a crucial role in plants response to abiotic stresses [39,40,41,42]. When plants are exposed to adverse environments, reactive oxygen species (ROS) accumulate in large quantities within the plant [43]. Melatonin is recognized as an efficient antioxidant that safeguards plants from oxidative damage by neutralizing excessive ROS within plants [44]. Previous studies found that exogenous melatonin enhances the antioxidant capacity and reduces ROS damage in apples, tomatoes, and wheat under drought stress [25,26,27]. Our results showed that melatonin seed coating helped to reduce the content of H_2_O_2_, and O_2_^•−^ in wheat seedlings (Figure 3), and their lower content might be caused by an increase in proline or other antioxidant contents. It is worth noting that Figure 3a showed that low concentrations of melatonin significantly inhibited the generation of H_2_O_2_; however, when melatonin rises to 100 µM, the content of H_2_O_2_ also significantly increased. This might be related to the fact that melatonin not only eliminates H_2_O_2_ but also induces its production when its concentration exceeds a certain level [45]. Additionally, melatonin enhanced the stress resistance of plants by elevating the activity of antioxidant enzymes. After being treated with melatonin, the wheat plants had a higher activity of SOD, CAT, and APX, which helped alleviate the damage caused by low temperature [46]. As shown in Figure 4, the results of this study also demonstrate that, under drought conditions, 100 µM melatonin significantly enhances the activities of CAT, Cu/Zn-SOD, POD, and T-GSH in wheat seedlings, thereby strengthening the stress resistance of wheat seedlings. Furthermore, 100 or 50 µM melatonin seed coating enhanced the contents of soluble proteins and soluble sugars of wheat seedlings (Figure 5a,b). Chlorophyll plays a crucial role in the growth and development of plants and their resistance to external stresses. Previous studies have shown that melatonin can promote the accumulation of chlorophyll by suppressing the expression of chlorophyll degradation genes [47]. Similarly, our results showed that compared with CK group, treatment with 200 µM melatonin seed coating significantly increased the contents of total chlorophyll in wheat (Figure 5c).

The application of melatonin in plants can notably enhance crop yields, mainly through increasing the weight of fruits. Previous study reported that spraying melatonin twice during the growing season to grapevines resulted in an approximately 6.6% increase in the weight of grape berries compared to CK [48]. This research further found that melatonin seed coating significantly increased the weight of mature wheat fruit, with a significant increase in both thousand seed weight and yield compared to CK group (Figure 6c–e). Additionally, melatonin treatment helped accumulate phenolic compounds in grapefruit, which makes the wine produced from fruits have a stronger fruit aroma and sweetness, enhanced the quality of red wine [49]. Likewise, this study also demonstrated that melatonin seed coating elevated protein and wet gluten contents of wheat grains, hence boosting the overall quality of wheat (Figure 7).

## 5. Conclusions

This study revealed that 100 µM exogenous melatonin significantly enhanced the germination rate of wheat seeds within 24 h, with an 11% increase compared to CK group. Under drought conditions, 100 µM melatonin seed coating was the most conducive to the growth of wheat, significantly elevated fresh weight, plant height, and root length of wheat seedlings. Further studies demonstrated that 100 µM melatonin seed coating significantly increased the activity of antioxidant enzymes (CAT, POD, Cu/Zn-SOD, T-GSH) in wheat seedlings. Likewise, an appropriate concentration of melatonin seed coating also inhibited the accumulation of reactive oxygen species (ROS) within wheat seedlings. Then, 100 µM melatonin seed coating also increased the contents of soluble protein and chlorophyll in wheat seedlings under drought conditions, further enhancing the resistance of wheat to drought stress. Moreover, compared to the CK group, 100 µM melatonin seed coating significantly improved the yield of wheat under drought conditions. In brief, melatonin seed coating is anticipated to become an effective approach for increasing the yield of wheat under drought conditions.

## Data Availability

Data will be made available on demand.

## Supplementary Materials

The following supporting information can be downloaded at: https://www.mdpi.com/article/10.3390/biology14010094/s1, Table S1: The physical and chemical properties of the soil; Table S2: The surface temperature of the wheat from sowing to harvest.
