# Supplementary material for: Melatonin Promotes Yield Increase in Wheat by Regulating Its Antioxidant System and Growth Under Drought Stress
Source: Biology (Basel). 2025 Jan 18;14(1):94. doi: 10.3390/biology14010094 (PMC11760888; doi:10.3390/biology14010094)
Supplement: Supplementary file 1 [file biology-14-00094-s001.zip › biology-3409385-supplementary.pdf]

Supplementary Materials for

Melatonin Promotes Yield Increase of wheat by Regulating Its Antioxidant System and Growth Under Drought Stress

The supplementary material contains 2 tables.

Table S1. The physical and chemical properties of the soil

Compound Name	Content
organic matter	43.2(g/kg)
total nitrogen	0.337%
total phosphorus	0.121%
total potassium	1.9%
water-soluble nitrogen	720 mg/kg
available phosphorus	183.5 mg/kg
available potassium	470 mg/kg
moisture	8.2%
pH	5.14

Table S2. The surface temperature of the wheat from sowing to harvest

date	Average High Temperature (°C)	Average low temperature (°C)
October 2023	23	14
November 2023	21	9
December 2023	18	7
January 2024	17	5
February 2024	20	7
March 2024	24	10
April 2024	28	14
